# Liquid biopsy-based tumor profiling for metastatic colorectal cancer patients with ultra-deep targeted sequencing

**DOI:** 10.1371/journal.pone.0232754

**Published:** 2020-05-07

**Authors:** Jun-Kyu Kang, Sunghoon Heo, Hwang-Phill Kim, Sang-Hyun Song, Hongseok Yun, Sae-Won Han, Gyeong Hoon Kang, Duhee Bang, Tae-You Kim

**Affiliations:** 1 Department of Molecular Medicine & Biopharmaceutical Sciences, Graduate School of Convergence Science and Technology, Seoul National University, Seoul, Republic of Korea; 2 Cancer Research Institute, Seoul National University, Seoul, Republic of Korea; 3 Department of Chemistry, Yonsei University, Seoul, Republic of Korea; 4 IMBDx Inc., Seoul, Republic of Korea; 5 Center for Precision Medicine, Seoul National University Hospital, Seoul, Republic of Korea; 6 Department of Internal Medicine, Seoul National University Hospital, Seoul, Republic of Korea; 7 Department of Pathology, Seoul National University Hospital, Seoul, Republic of Korea; CNR, ITALY

## Abstract

Analyzing cell-free DNA (cfDNA) as a source of circulating tumor DNA is useful for diagnosing or monitoring patients with cancer. However, the concordance between cfDNA within liquid biopsy and genomic DNA (gDNA) within tumor tissue biopsy is still under debate. To evaluate the concordance in a clinical setting, we enrolled 54 patients with metastatic colorectal cancer and analyzed their plasma cfDNA, gDNA from peripheral blood mononuclear cells (PBMC), and gDNA from available matched tumor tissues using ultra-deep sequencing targeting 10 genes (38-kb size) recurrently mutated in colorectal cancer. We first established a highly reliable cut-off value using reference material. The sensitivity of detecting KRAS hotspot mutations in plasma was calculated as 100%, according to digital droplet PCR. We could selectively detect clinically important somatic alterations with a variant allele frequency as low as 0.18%. We next compared somatic mutations of the 10 genes between cfDNA and genomic DNA from tumor tissues and observed an overall 93% concordance rate between the two types of samples. Additionally, the concordance rate of patients with the time interval between liquid biopsy and tumor tissue biopsy within 6 months and no prior exposure to chemotherapy was much higher than those without. The patients with KRAS mutant fragments in plasma had poor prognosis than those without the mutant fragments (33 months vs. 63 months; p<0.05). Consequently, the profiling with our method could achieve highly concordant results and may facilitate the surveillance of the tumor status with liquid biopsy in CRC patients.

## Introduction

Circulating tumor DNA (ctDNA) can be detected in the plasma as cell-free DNA (cfDNA) in patients with cancer because of various biologic processes, including cell necrosis, apoptosis, and exosomal transport [1,2]. Thus, genomic profiling of cfDNA with liquid biopsy has been developed for diagnosing and monitoring patients with cancer [3]. Among the many available sequencing methods, next-generation sequencing (NGS), unlike biased molecular tests, allows comprehensive analysis of genomes [4]. Because of its high sensitivity for detection and multiplexed interpretation, NGS is suitable for non-invasive profiling of cancers using cfDNA [5]. Targeted exome sequencing of cfDNA is a low-cost NGS technique that can detect gene mutations specific to the target treatment. For example, acquired resistance to cetuximab in patients with colorectal cancer has been monitored using liquid biopsies and NGS technology [6]. A non-invasive method for profiling genetic alterations in cfDNA from patients with cancer may provide valuable information both before and after diagnosis.

A recent study used deep sequencing targeting 508 genes to evaluate plasma cfDNA and matched tumor tissues from 124 patients with metastatic cancer. The results showed a concordance rate of only 84% among patients, and cfDNA of unknown origin accounted for 45% of all samples [7]. This discrepancy may be attributed to the types of samples or analysis platform. For example, when comparing detection rates of endothelial growth factor receptor(EGFR) T790M mutations between tumor tissue and plasma, the sensitivity and specificity were 77% and 63%, respectively, using digital droplet PCR (ddPCR) [8]. Likewise, when using two different analysis platforms for the same samples, the concordance rate was only 22.5% (9 of 40 patients), indicating only partial concordance between platforms [9]. Furthermore, the concordance rate between two commercial platforms (Guardant360 for plasma and Foundation One for tumor tissues) was 64.7% and 88.2% when the time interval between obtaining the two types of samples was >0.8 months versus ≤0.8 months [10]. Thus, discordance between genetic profiling of tumor tissues and plasma cfDNA has interfered with the use and interpretation of liquid biopsy-based assays.

In this study, we devised a simple bioinformatics pipeline for discovering somatic mutations, which removes sequencing errors from true mutation signals during ultra-deep targeted sequencing of cfDNA from patients with cancer. We focused on developing a feasible method for profiling the plasma of patients with metastatic colorectal cancer and evaluating reasons for discrepancies between genetic profiles obtained from cfDNA and tumor tissue samples that may be relevant in the clinical setting.

## Material and methods

### Patient cohort and ethics statement

In this study, we retrospectively reviewed clinical data of 54 patients with metastatic colorectal cancer who underwent surgery or chemotherapy at Seoul National University Hospital, South Korea. We collected clinicopathologic information, including sex, age, pathologic data, and therapeutic information (Table 1). Fifty-four blood samples were collected between January 2014 and February 2015 at our institution. All patients provided written informed consent before any study-specific procedures, including liquid biopsy, tissue biopsy, and genetic testing. The study protocol was approved by the Institutional Review Board (IRB) of Seoul National University Hospital [IRB number: 1407-060-592], and the study was conducted in accordance with the recommendations of the Declaration of Helsinki for biomedical research involving human subjects.

**Table 1:** Characteristics of patients with metastatic colorectal cancer (N = 51).

Clinicopathologic feature		Value
Age, y		62 (26–76)
Sex	Male	32 (62.74)
	Female	19 (37.25)
Primary tumor site	Proximal colon	10 (19.61)
	Distal colon	23 (45.09)
	Rectum	18 (35.29)
Metastasis site	Liver	26 (50.98)
	Lungs	20 (39.21)
	Peritoneum	14 (27.45)
	Lymph nodes/other organs	22 (43.13)
Treatment regimen at the time of liquid biopsy	None (before chemotherapy)	27 (52.94)
	FOLFOX	11 (21.57)
	FOLFIRI	6 (11.76)
	FOLFIRI + bevacizumab	1 (1.96)
	Capecitabine	6 (11.76)
Time interval between tissue and liquid biopsies	≤6 months	26 (50.98)
	>6 months	25 (49.02)

Data are median (range) or number (percentage).

### Tumor tissue samples

We used archival tissue samples from patients included in the study, when available. Among 40 available matched tumor specimens, 24 were formalin-fixed, paraffin-embedded (FFPE) tissues, and 16 were fresh-frozen tissues. Genomic DNA was isolated from each sample using a Qiagen DNA FFPE Tissue Kit (Qiagen, Hilden, Germany) for FFPE samples and a QIAamp DNA Mini Kit (Qiagen) for fresh-frozen tissues. After isolation, the concentrations and purities of genomic DNA were measured using a spectrophotometer (ND1000; Nanodrop Technologies, Thermo Fisher Scientific, MA, USA).

### Blood samples and cell-free DNA isolation and quantification

Whole blood (8–10 mL) was collected into EDTA tubes during routine phlebotomy from patients who had volunteered to donate blood samples for research purposes. Blood samples were centrifuged with Ficoll solution at 1,500 × g for 15 min. Plasma was then separated by centrifugation at 16,000 × g for 10 min to remove cell debris, after which 1-mL aliquots were placed in Eppendorf tubes and stored at −80°C before extraction. This protocol was performed within 20 min of blood collection to prevent cfDNA degradation.

cfDNA was isolated according to the manufacturer’s instructions from 2–4 mL plasma using a QIAamp Circulating Nucleic Acid Kit (Qiagen) with the QIAvac 24 Plus vacuum manifold and quantified using a 2200 TapeStation (Agilent Technologies, Santa Clara, CA, USA). Peripheral blood mononuclear cells (PBMC) were separated following this protocol. Genomic DNA was isolated from PBMC using a QIAamp DNA Mini Kit (Qiagen).

### Ultra-deep targeted sequencing and variant calling

Samples from patients with metastatic colorectal cancer were analyzed using ultra-deep targeted sequencing for a panel of 10 genes recurrently mutated in this cancer: KRAS, TP53, APC, BRAF, PIK3CA, SMAD4, ATM, ARID1A, ACVR2A, and TCF7L2 [11–13]. A DNA NGS library was constructed using a Celemics NGS DNA Library Prep Kit. For cfDNA, a random barcode was introduced into P7 index sites to recover more reads, which were assumed to be PCR duplicates based on a previous analytic method [14]. Solution-based target enrichment was performed at Celemics, Inc. (South Korea), using a custom target capture panel. Captured DNA libraries were sequenced using an Illumina HiSeq 2500 platform (Illumina, San Diego, CA, USA) in 2 × 150 bp paired-end mode. Filtered fastq files were aligned to the hg19 reference genome using Burrows–Wheeler Aligner (v0.7.10) “mem” algorithm. Aligned SAM files were converted into BAM files and sorted using SAMtools (v1.1). PCR duplicates were removed with Picard tools (v1.115) “MarkDuplicates” algorithm. Local realignment around known indel sites and base quality score recalibration were performed with GATK (v4.1.0.0). After generating pileup files with SAMtools mpileup, variants were called using VarDict [15]. Genetic variants were annotated using ANNOVAR (v2016-02-01) [16] and other in-house scripts. For hotspot mutations, we screened and rescued all positions indicating clinically important somatic mutations based on COSMIC [17] and My Cancer Genome [18] using Integrative Genomics Viewer [19].

### Raw data filtering

We analyzed ctDNA reference material (HD780, Horizon Dx) to set limits of detection. These materials were analyzed by our panel for every condition (wild-type [WT], 5%, 1%, and 0.1%). The specificity and sensitivity were 100% for WT, 5%, and 1%, but the sensitivity was lower for 0.1%. Through this step, our platform could detect four minimum alteration alleles in 400× coverage, systemically. Samples that did not reach this parameter of coverage after deduplication were excluded. Three plasma samples and 13 FFPE samples were excluded because of poor NGS quality.

### Validation of circulating mutant fragments

Using ddPCR, we validated KRAS mutant fragments in a hotspot (G12D, G12V, and G13D) in plasma. cfDNA was isolated from 2 mL plasma collected and analyzed by ddPCR (QX200 ddPCR system; Bio-Rad, Berkeley, CA, USA), in accordance with the MACROGEN, Inc., protocol. Because of the limited availability of plasma, only the most common KRAS (i.e., G12D, G12V, and G13D) mutations were analyzed. The proportion of mutant KRAS alleles (i.e., fractional abundance) was calculated as follows: drops positive for the mutant allele / (drops positive for the mutant allele + drops positive for the WT allele).

### Concordance analysis

The types of mutations detected in cfDNA, PBMC, and tumor tissue were identified as single nucleotide variants (SNVs) and insertions or deletions (InDels). For the 10 genes, concordance was calculated using ultra-deep targeted sequencing data of tumor tissue as the gold standard. All positions indicating mutations were screened, except for synonymous mutations. Mutations in the same positions in PBMC as in cfDNA or tumor tissues were considered germline mutations, whereas mutations not detected in PBMC were considered somatic mutations. Mutations detected in cfDNA were compared with mutations detected in tumor tissue with a variant allele frequency (VAF) more than 1%; when detected in both samples, these mutations were considered concordant.

### Analysis of metastatic lesions

Patients included in this study had metastatic lesions in the liver, lungs, peritoneum, or other organs. Metastatic tumor burdens in the liver and lungs were estimated by calculating the sum of the longest diameter of the tumors visualized on abdominal and chest computed tomography (CT) scans.

### Statistical analysis

Two-sided χ^2^ tests were used to compare categorical variables, and Student’s t-tests were used to compare continuous variables. Spearman’s correlation coefficients (ρ) were calculated using PRISM software (GraphPad, La Jolla, CA, USA). Other statistical tests were performed using R, version 3.2.5 (http://www.r-project.org). Kaplan-Meier curves were constructed to analyze overall survival, and subgroups were compared using log-rank tests. P-values <0.05 were considered statistically significant.

## Results

### Patient characteristics

Of the 54 patients with metastatic colorectal cancer screened for enrollment in this study, 51 patients were included after raw data quality control (QC). The primary tumor sites were the proximal colon (n = 10), distal colon (n = 23), and rectum (n = 18). Metastatic lesions were present in the liver (n = 26), lungs (n = 20), peritoneum (n = 14), and lymph nodes/other organs (n = 22) (Table 1). In 27 of the 51 patients, the liquid biopsy was obtained no prior exposure to chemotherapy, and in the remaining patients, this biopsy was obtained after postoperative chemotherapy (FOLFOX, FOLFIRI, FOLFIRI-bevacizumab, or capecitabine). The time interval between liquid and tissue biopsies was ≤6 months in 29 of the 51 patients and >6 months in 22 patients. For each patient, we isolated DNA from plasma, PBMC, and matched tumor tissues for ultra-deep targeted panel sequencing.

### Population frequency–based panel design and methodologic optimization

**Fig 1:**
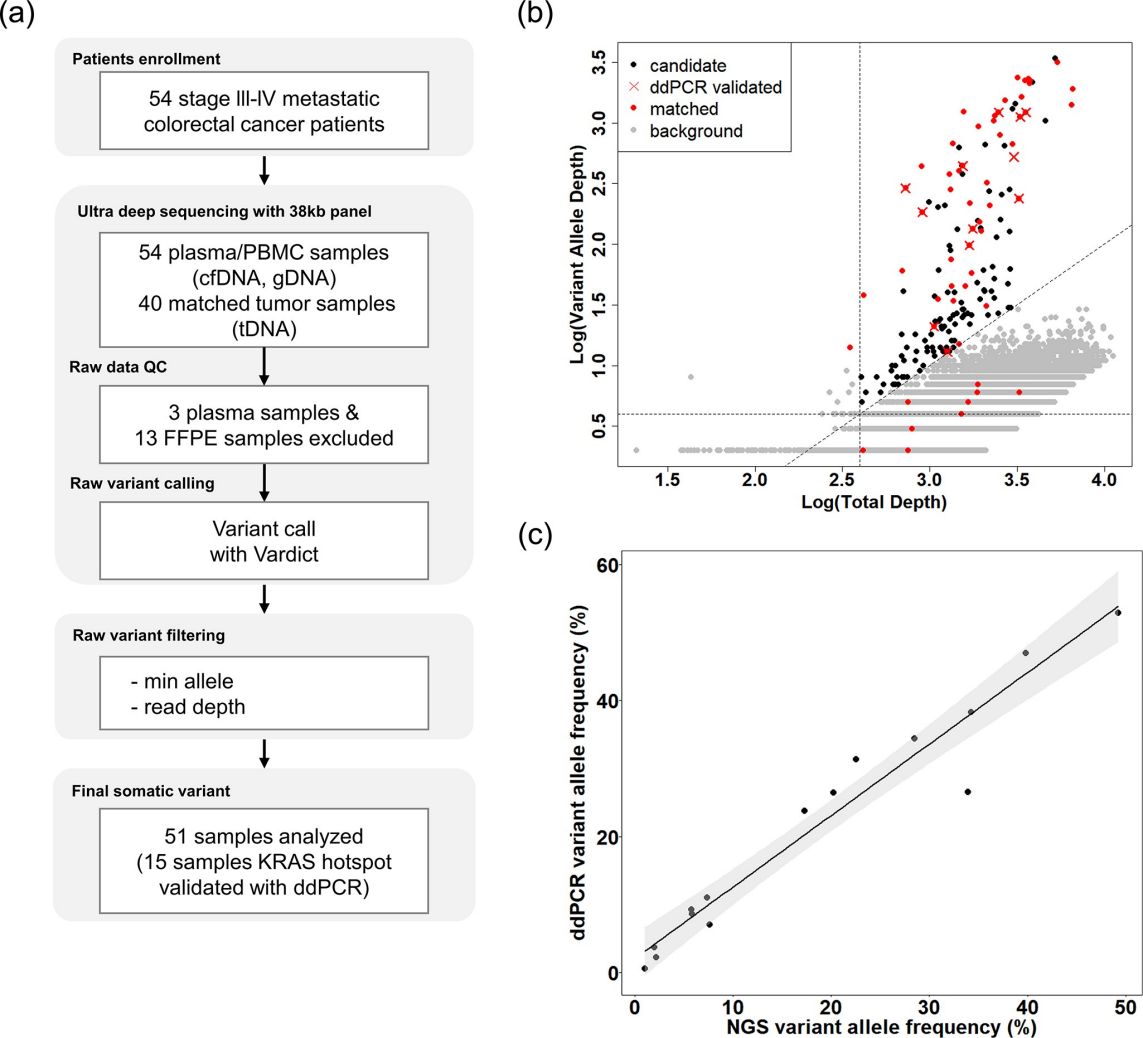
Complete NGS workflow for analysis of cfDNA, tumor tissue DNA, and PBMC DNA from patients with metastatic colorectal cancer. (A) Workflow for analysis of cfDNA and gDNA from tumor tissues and PBMC. (B) Analysis of biologic background in all somatic variants of plasma cfDNA. cfDNA variants concordant with matched tissue are shown in red. cfDNA variants validated with the ddPCR platform are indicated with an ‘X’. (C) Correlation between NGS and ddPCR results of cfDNA VAF (n = 15, R^2^ = 0.94, Pearson’s ρ = 0.97). cfDNA, cell-free DNA; ddPCR, droplet digital polymerase chain reaction; FFPE, formalin-fixed, paraffin-embedded; gDNA, genomic DNA; NGS, next-generation sequencing; PBMC, peripheral blood mononuclear cells; VAF, variant allele frequency.

Our panel for ultra-deep targeted sequencing contains diagnostically important mutated genes in colorectal cancer that are highly mutated among patients with this malignancy: APC, TP53, KRAS, BRAF, PIK3CA, SMAD4, ATM, ARID1A, ACVR2A, and TCF7L2 [11–13]. Coverage of these genes was more than 99% in other tissue-based cohorts in publicly available databases (TCGA, MSKCC, GENIE; S1 Fig) [20–22]. To verify the performance of our platform, we conducted ultra-deep targeted sequencing with reference materials harboring mutant fragments (e.g., KRAS p.G12D, PIK3CA p.E545K) validated by ddPCR in various conditions. With this procedure, we confirmed that our platform can perfectly detect a VAF of 1% (S2 Fig). Next, we conducted ultra-deep targeted sequencing with plasma cfDNA, as well as gDNA from PBMC and available matched tumor tissues from 54 patients with colorectal cancer. Samples with low quality sequencing data were excluded in the pre-QC step. We obtained a median sequencing depth of 1,149× for cfDNA, 1,533× for tumor tissue, and 1,177× for PBMC after deduplication and were able to detect mutant fragments with a VAF as low as 0.18% (S3 Fig, Table 2). True variants were then detected based on the cut-off value. Finally, cfDNA with KRAS hotspot mutations was validated using ddPCR (Fig 1A). In the optimization step, somatic variants with <1% VAF were excluded, and variant candidates were sorted from 69,081 to 175. Somatic variants validated by ddPCR were included in the range of candidates. Nine actionable somatic variants present in the systemic background noise were rescued manually (Fig 1B). The panel sequencing results were validated using the ddPCR platform to detect mutations at the KRAS hotspots (codons 12 and 13 of exon 2). Results obtained with the panel sequencing platform and the ddPCR platform corresponded perfectly (100%), and results of each NGS platform were positively correlated (n = 15, R^2^ = 0.94, Spearman’s ρ = 0.97; Fig 1C). Thus, the performance of ultra-deep targeted sequencing with appropriate variant calling was easily optimized for detecting circulating cfDNA variants.

**Table 2:** Summary of 10 genes-targeted sequencing.

Summary of targeted deep sequencing	
**Percentage of plasma samples with at least one mutation in cell-free DNA when tissue mutation present**	
96.00%	
**Mean value of on-target coverage for each sample types**	
**cell-free DNA**	1,149.63
**Tumor tissue**	1,533.98
**PBMC**	1,177.68
**Range of detected variant allele frequencies in cell-free DNA**	
0.18%-78.39%	

**Concordance between liquid biopsy and tumor biopsy NGS results**

**Fig 2:**
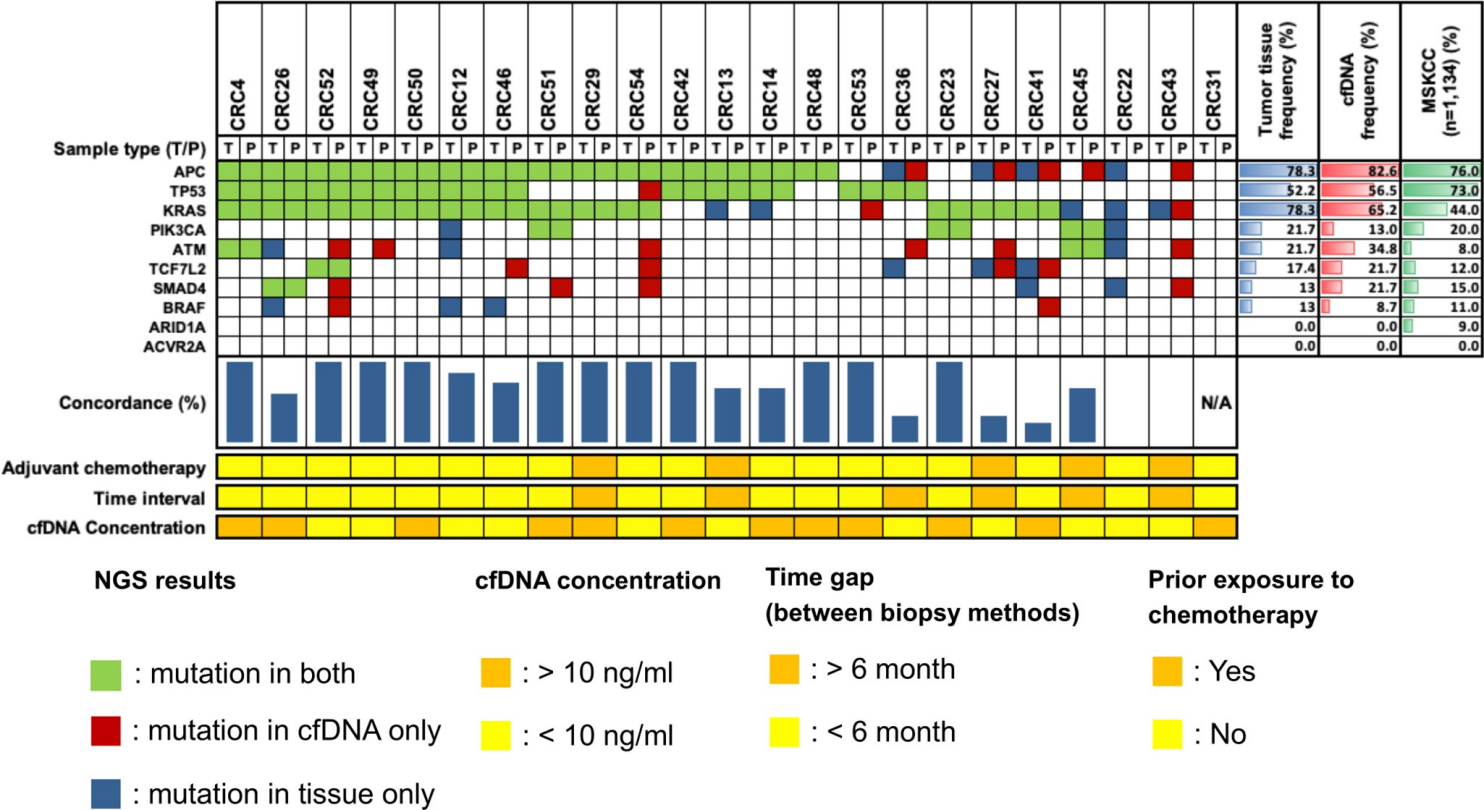
Actionable cfDNA mutations detected by ultra-deep targeted sequencing. Somatic variants are marked in red (cfDNA only), blue (tumor tissue only), and green (both cfDNA and tumor tissue). Population frequencies for each sample type are shown as red (cfDNA) and blue (tumor tissue) gradients. Concordance rates are indicated by vertical blue bars. cfDNA, cell-free DNA; N/A, not applicable; NGS, next-generation sequencing; P, plasma; T, tumor tissue.

Concordance of ultra-deep targeted sequencing results was estimated in samples from 51 patients with available cfDNA and genomic DNA from PBMC and primary tumor tissues. Genomic profiling was conducted in 23 patients with matched tumor tissues and cfDNA (Fig 2). APC, KRAS, and TP53 were the mutated genes most frequently detected in plasma cfDNA. They were also the most frequently detected mutated genes in tumor tissues, as well as in a colorectal cancer database (MSKCC; n = 1,134). The overall median concordance rate between cfDNA and tumor tissue DNA was 93% among all patients. One patient (CRC31) exhibited no mutation in either plasma cfDNA or tumor tissue. The concordance rates for each gene were as follows: TP53, 100%; APC, 91.3%; and KRAS, 78.3%.

**Fig 3:**
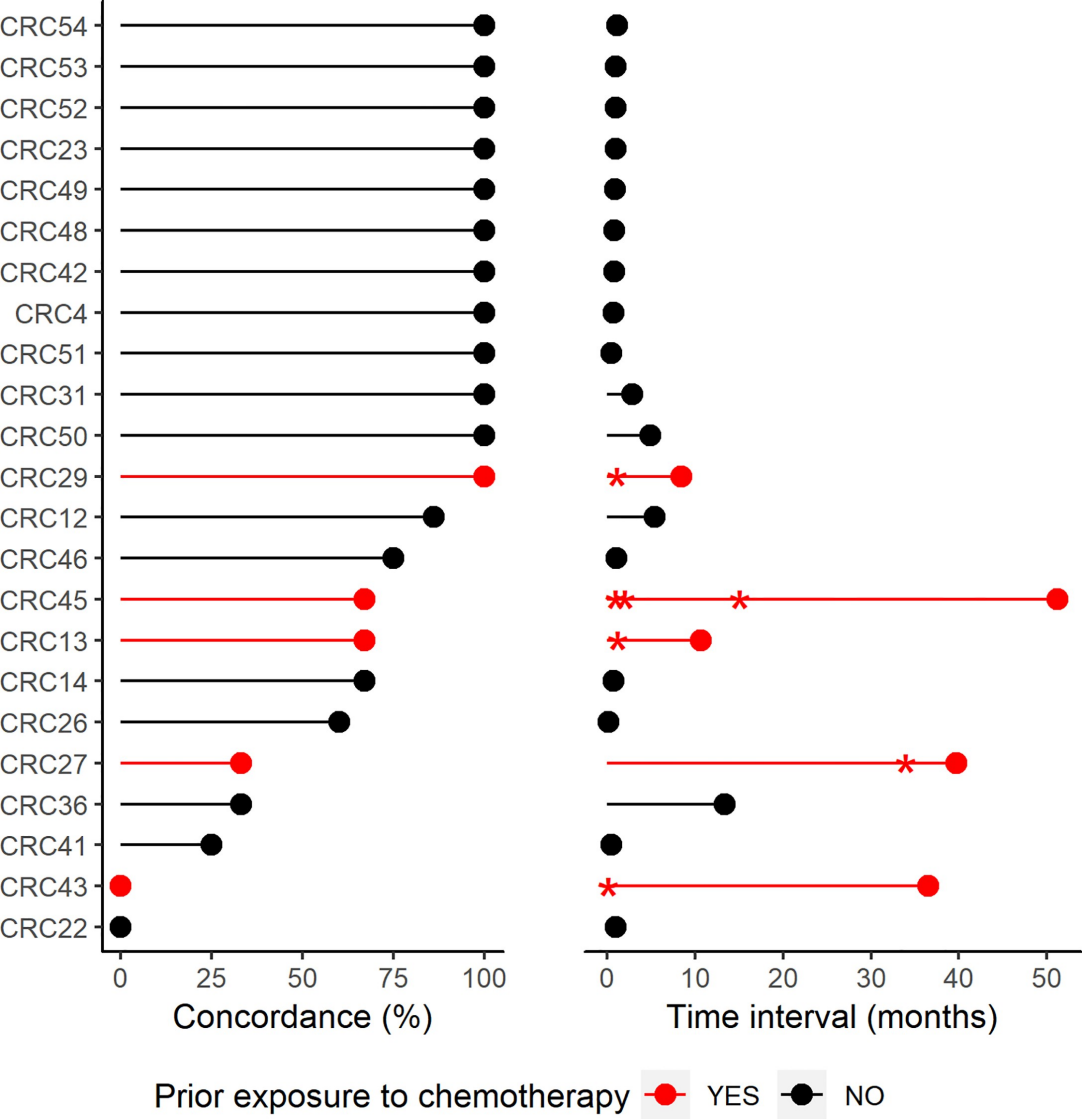
Concordance rates between cfDNA and tumor tissue DNA for the evaluated mutant genes according to the time interval between samples and chemotherapy treatment status (n = 23). Patients with prior exposure to chemotherapy were indicated with red color. Concordance rate for individual were plotted on left panel. Time interval between tissue biopsy and liquid biopsy were plotted on right panel. And time points of chemotherapy are indicated with asterisks.

Next, we evaluated clinical factors that may affect concordance. According to a previous report, the time interval between liquid and tissue biopsy is one of the most important factors affecting concordance between plasma cfDNA and primary tumor tissue DNA [23]. In 6 of 23 patients (26%), the interval between liquid and tissue biopsies was 6 months. The average concordance rate was 50.0% in these patients, and 83.1% in patients with a ≤6 month-interval. In addition, the concordance rate was 80.3% in chemotherapy-naive patients and 53.4% in patients evaluated while receiving chemotherapy (Fig 3). Furthermore, the mean concordance rate was 60.1% in patients with a lower cfDNA concentration (≤10 ng/mL) and 87.6% in patients with a higher concentration (>10 ng/mL). Thus, for the 10 targeted genes, plasma samples with a higher cfDNA concentration, collected within 6 months of primary tumor biopsy, and obtained from patients prior to initiation of chemotherapy may be especially likely to represent the primary tumor.

### Analytic estimation for cfDNA assay

**Fig 4:**
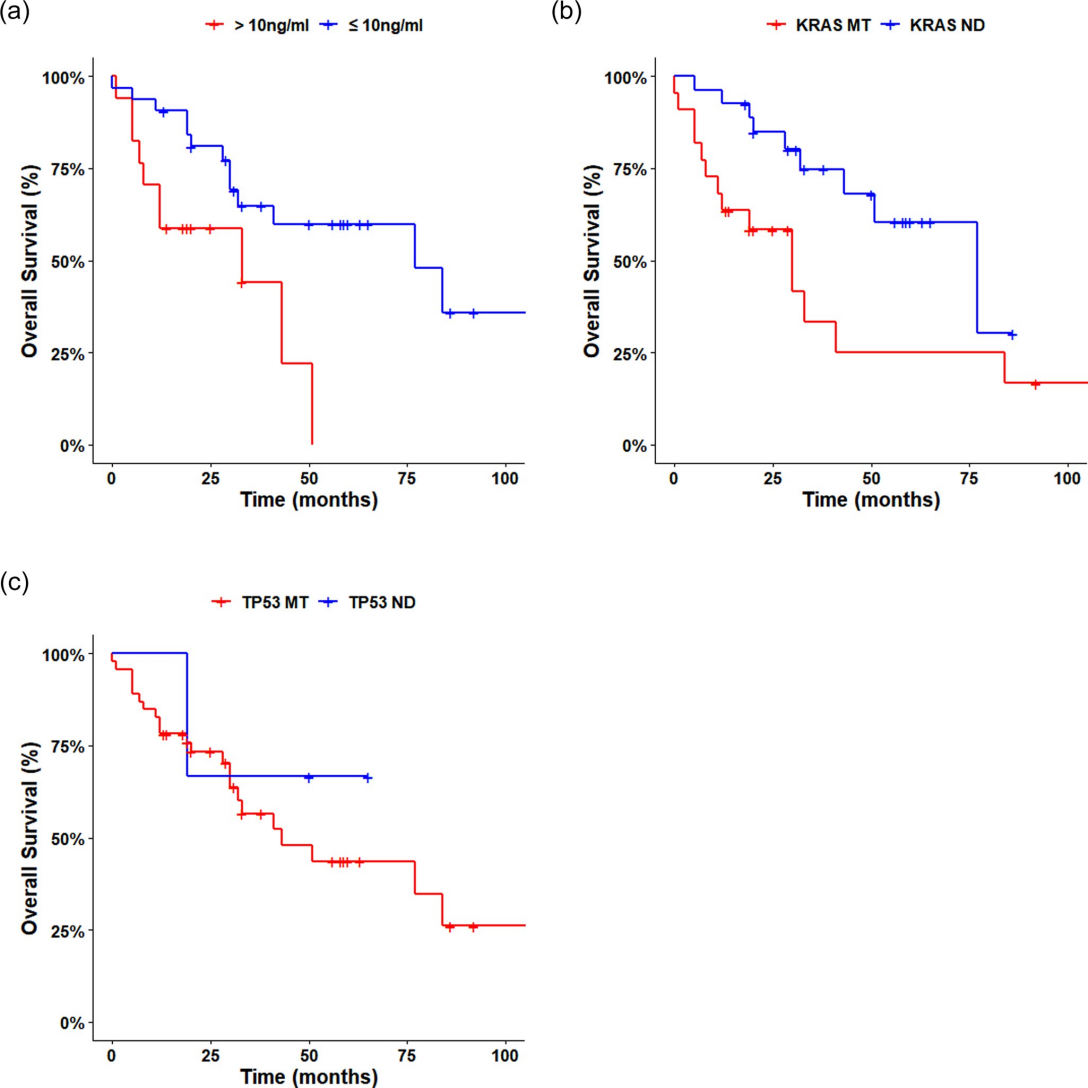
Survival of patients with actionable mutant fragments detected in plasma. (A) Overall survival according to whether KRAS mutant fragments were or were not detected in baseline plasma cfDNA (n = 51, p<0.05). (B) Kaplan-Meier curves for overall survival in patients according to cfDNA concentration. Survival was worse in patients with higher cfDNA concentrations (>10 ng/mL, n = 17) than in those with lower cfDNA concentrations (≤10 ng/mL, n = 34) (log-rank test, p<0.05). (C) Kaplan-Meier curves for overall survival according to whether cfDNA TP53 mutants were detected. Overall survival was worse in patients with TP53 mutants, although this did not reach statistical significance (Log-rank test, p<0.1). cfDNA, cell-free DNA; MT, mutant detected; ND, mutant not detected.

As KRAS is one of the most important mutated genes in colorectal cancer, we hypothesized that patients harboring KRAS mutant fragments may have a poor clinical phenotype. Overall survival was worse in patients with higher cfDNA concentrations or with oncogenic mutant fragments detected in the plasma than in patients with lower cfDNA concentrations or without plasma oncogenic mutant fragments, confirming the association of these factors with a poorer prognosis (Fig 4). In particular, median survival was 33 months in patients with KRAS mutant fragments and 63 months in patients without these fragments (n = 51, Log-rank test, p<0.05). In addition, median survival was 33 months in patients with high concentration cfDNA and 77 months in patients with low concentration cfDNA (n = 51, Log-rank test, p<0.05). median survival was 43 months in patients with TP53 mutant fragments and NA in patients without these fragments (n = 51, Log-rank test, p<0.1). We also determined the concordance between the results of cfDNA NGS and the results of Sanger sequencing (codons 12 and 13 of KRAS exon 2) in the available tumors. Using the Sanger sequencing results as the gold standard, the sensitivity of cfDNA NGS was calculated to be 86.36% (n = 48, two-sided χ^2^ test, p = 0.0003) (S1 Table).

To demonstrate the clinical utility of our approach, we explored whether tumor volume affected the quantity of tumor-derived cfDNA. We selected patients with measurable liver metastases to reliably assess systemic tumor volume because liver lesions were more completely and reproducibly measurable on contrast-enhanced CT scans than other metastatic lesions. Furthermore, the liver is the most common site of metastatic lesions for gastrointestinal cancers [24]. Metastatic liver lesions were detected in 26 of 51 patients. For each patient, we calculated the sum of the longest diameters of all liver lesions detected by CT. The results showed a positive correlation between plasma levels of mutated KRAS fragments and burden of metastatic liver lesions (n = 12, R^2^ = 0.55, Spearman’s ρ = 0.69). A positive correlation was also observed between metastatic burden in the liver and plasma KRAS VAF (S4 Fig). Meanwhile, among patients with at least one metastatic liver lesion, the results of the samples were analyzed according to the time interval between liquid and tumor tissue biopsies (S2 Table). The concentration of cfDNA was positively correlated with the fraction of ctDNA. In addition, KRAS VAF (%) correlated positively with liver metastatic burden. Overall, these results suggest that KRAS mutant fragments in plasma can reflect the status of patients with metastatic colorectal cancer.

## Discussion

In this study, we used a 38-kb panel targeting frequently mutated genes in colorectal cancer and performed ultra-deep targeted sequencing in all samples to detect somatic mutations with a low VAF [25]. Our platform reached an average of approximately 1,150× for cfDNA samples. Based on our deep sequencing coverage, variants with a VAF as low as 0.087% could be theoretically detected; however, the true VAF was <1.0%, which was our cut-off value surrounded by background errors. In our optimization step, we could suppress background error by 99.8%. This approach allowed us to detect mutation fragments (VAF 1.0%), which were validated by ddPCR. Other studies have used barcode sequencing to suppress NGS errors and detect very rare mutant fragments [26,27]. Therefore, molecular barcoding could be applied to our panel sequencing platform in further studies.

Genomic profiling of cfDNA has been used for diagnosing cancer and monitoring tumor progression [28]. In this study, we focused on estimating the concordance between tumor tissue DNA and cfDNA. Among 23 patients with available primary tumor tissue and cfDNA, the overall median concordance rate between samples was 93%, indicating that cfDNA did not perfectly reflect the DNA within the primary tumor (Figs 2 and 3). When exploring possible reasons for discrepancies, we found lower concordance in patients with a longer (>6 months) time interval between tissue and liquid biopsies than in those with a shorter interval (50.0% vs. 83.1%). Among the 17 patients with a ≤6-month time interval, cfDNA did not perfectly match the primary tumor tissue DNA in six patients. There are a few potential explanations for these discordant results. In one patient (CRC12), the time interval was 5 months. In four patients (CRCR14, CRC22, CRC26, CRC41, and CRC46), the liquid biopsy was obtained within 1 month after surgery, and the quantity of ctDNA has been previously demonstrated to be decreased after surgery [29]. The mean concordance rate of these four cases was 45.4%. Another five patients (CRC13, CRC27, CRC29, CRC43, and CRC45) received chemotherapy before the liquid biopsy was obtained. Chemotherapy may have promoted clonal selection in these patients, whose mean concordance rate was 53.4%. Therefore, the disconcordant results may have been affected by the time at which the liquid biopsies were obtained or by chemotherapy-induced intratumor genetic heterogeneity leading to biased tumor tissue results [6, 23, 30].

cfDNA profiling can reveal the presence of metastatic liver lesions more effectively than metastases in other organs because of the anatomical characteristics of the liver [24]. In addition, metastatic lesions in other organs are not as accurately quantified as liver metastases. Thus, because only KRAS fragments in plasma were validated with ddPCR, we hypothesized that KRAS mutant fragments in plasma, as well as the concentration of cfDNA, would be correlated with tumor burden in the liver. cfDNA concentrations were higher in patients with liver metastases than in patients with metastases in other organs, although the difference did not reach statistical significance (S4 Fig). Patients with metastatic liver lesions also had higher plasma levels of KRAS, APC, and TP53 mutant fragments, compared with patients without liver metastases, although statistical significance was achieved only for the KRAS fragments (S5 Fig). We also observed positive trends between ctDNA fraction and the extent of liver tumor burden (n = 12). Larger cohorts are required to validate these results. Our novel findings suggest that circulating fragments of mutant DNA in plasma may reflect the progression of liver metastases in advanced colorectal cancer and thereby represent a useful, non-invasive biomarker.

In conclusion, the current study showed that use of optimized somatic calling may allow detection of clinically actionable somatic mutations in plasma ctDNA.

## Supporting information

S1 Fig Frequently mutated genes in patients with colorectal cancer. Coverage was calculated in various large cohorts. Data for our previous study is shown in reference [20–22]. GENIE, the Genomics, Evidence, Neoplasia, Information, Exchange; MSKCC, Memorial Sloan Kettering Cancer Center; TCGA, The Cancer Genome Atlas.
(TIFF)

S2 Fig Setting the limit of detection with reference material for circulating tumor DNA. (A) List of shared genes between our panel and reference material (HD780). (B) Performance of our small-sized panel. Each dot represents variants detected using our ultra-deep targeted sequencing procedure.
(TIFF)

S3 Fig On-target coverage of ultra-deep targeted sequencing. On-target coverage of each sample was plotted before (A) and after (B) deduplication, using the PICARD tool. cfDNA, cell-free DNA; dedup, deduplication; FFPE, formalin-fixed, paraffin-embedded tumor tissue; fresh, fresh-frozen tumor tissue; pbmc, peripheral blood mononuclear cells.
(TIFF)

S4 Fig Correlation between plasma KRAS variant allele frequency and liver metastatic disease burden. Correlation between KRAS variant allele frequency and total size of liver metastases, excluding patients with disseminated metastatic lesions (n = 12, R^2^ = 0.55, Spearman’s ρ = 0.69).
(TIFF)

S5 Fig cfDNA concentrations and quantities of specific mutant fragments in plasma according to liver metastasis status. Comparisons between patients with liver metastasis and metastasis in other organs with respect to cfDNA concentration (A) and quantities of specific mutant fragments: KRAS (B), APC (C), and TP53 (D). The quantity of KRAS mutant fragments was significantly higher in patients with liver metastasis (Student’s t-test, p<0.05). ns, not significant.
(TIFF)

S1 Table Concordance of KRAS hotspots between plasma and tumor tissues. Hotspots were identified in tumor tissues by Sanger sequencing, which was considered the gold standard. Two-sided χ^2^ tests were used to compare categorical variables (p = 0.0003).
(XLSX)

S2 Table Concordance of quantifiable factors from targeted sequencing. (XLSX)
