# Supplementary figures and images for: Liquid biopsy-based tumor profiling for metastatic colorectal cancer patients with ultra-deep targeted sequencing
Source: PLoS One. 2020 May 7;15(5):e0232754. doi: 10.1371/journal.pone.0232754 (PMC7205246; doi:10.1371/journal.pone.0232754)

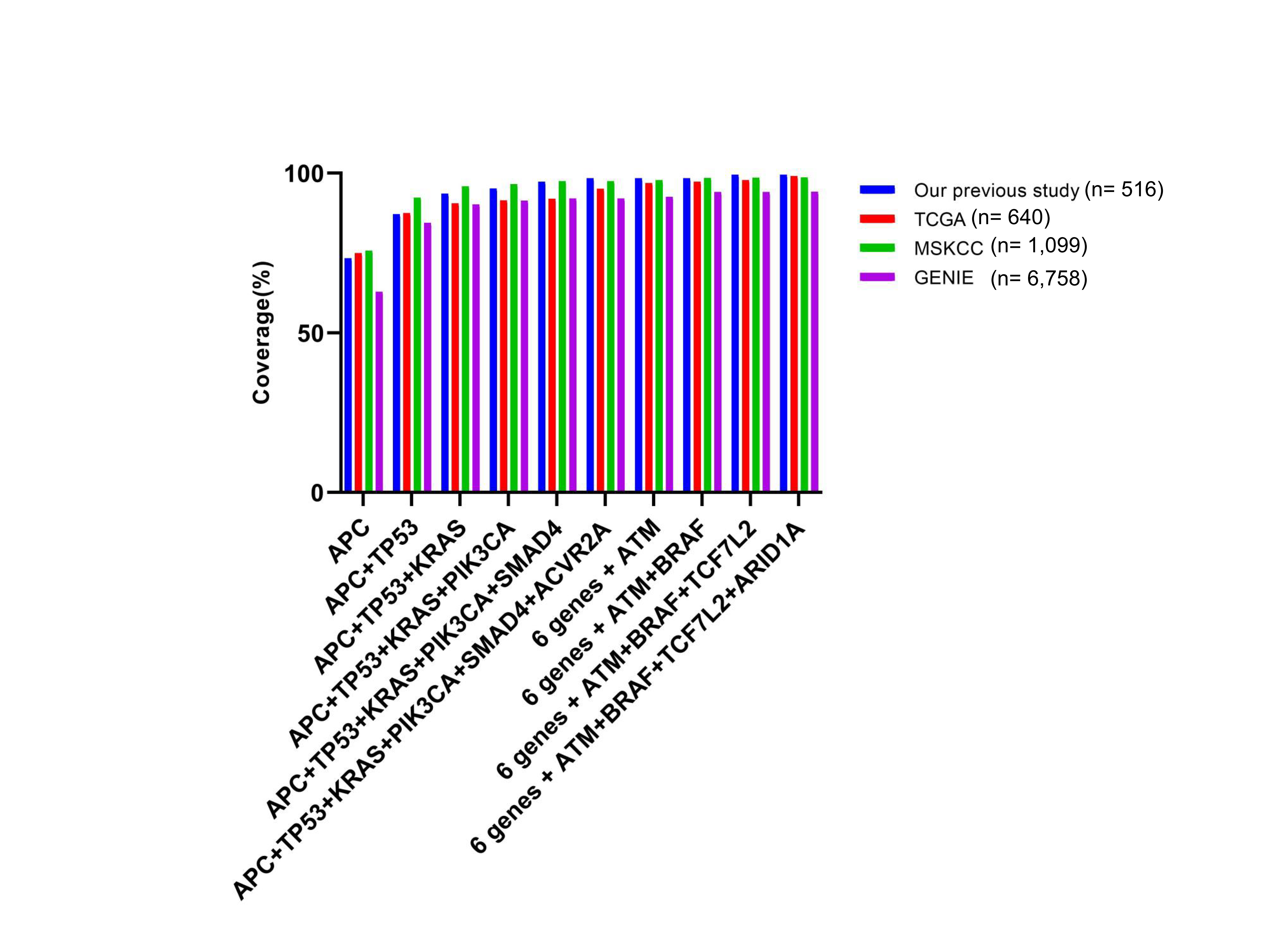

Supplement: S1 Fig — Coverage was calculated in various large cohorts. Data for our previous study is shown in reference [20–22]. GENIE, the Genomics, Evidence, Neoplasia, Information, Exchange; MSKCC, Memorial Sloan Kettering Cancer Center; TCGA, The Cancer Genome Atlas. (TIFF) [file pone.0232754.s001.tiff]

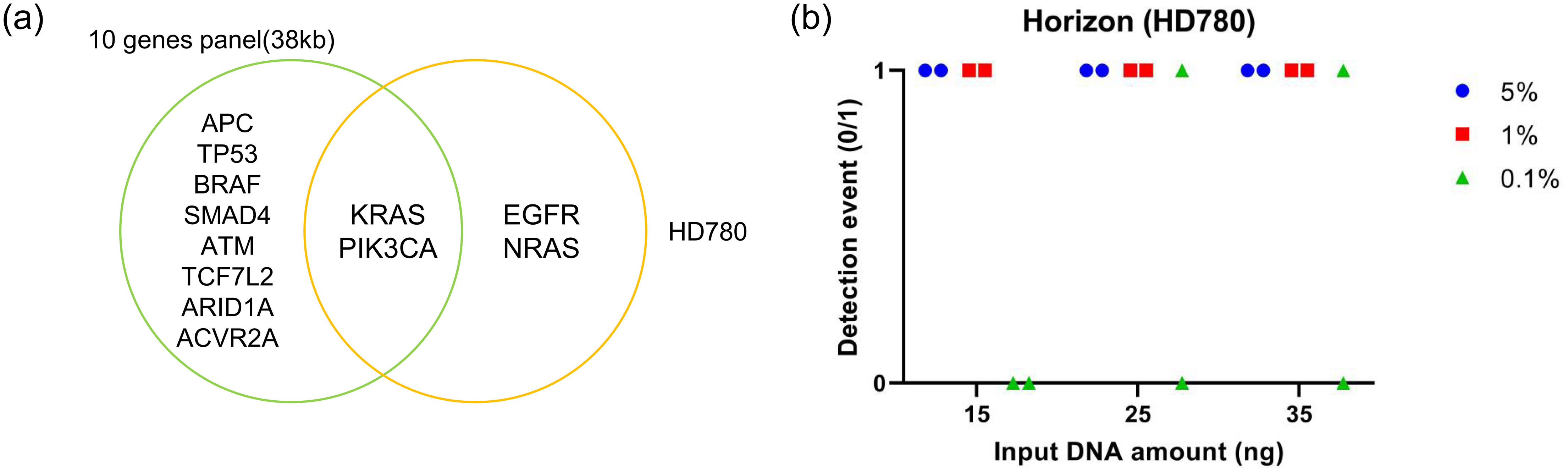

Supplement: S2 Fig — (A) List of shared genes between our panel and reference material (HD780). (B) Performance of our small-sized panel. Each dot represents variants detected using our ultra-deep targeted sequencing procedure. (TIFF) [file pone.0232754.s002.tiff]

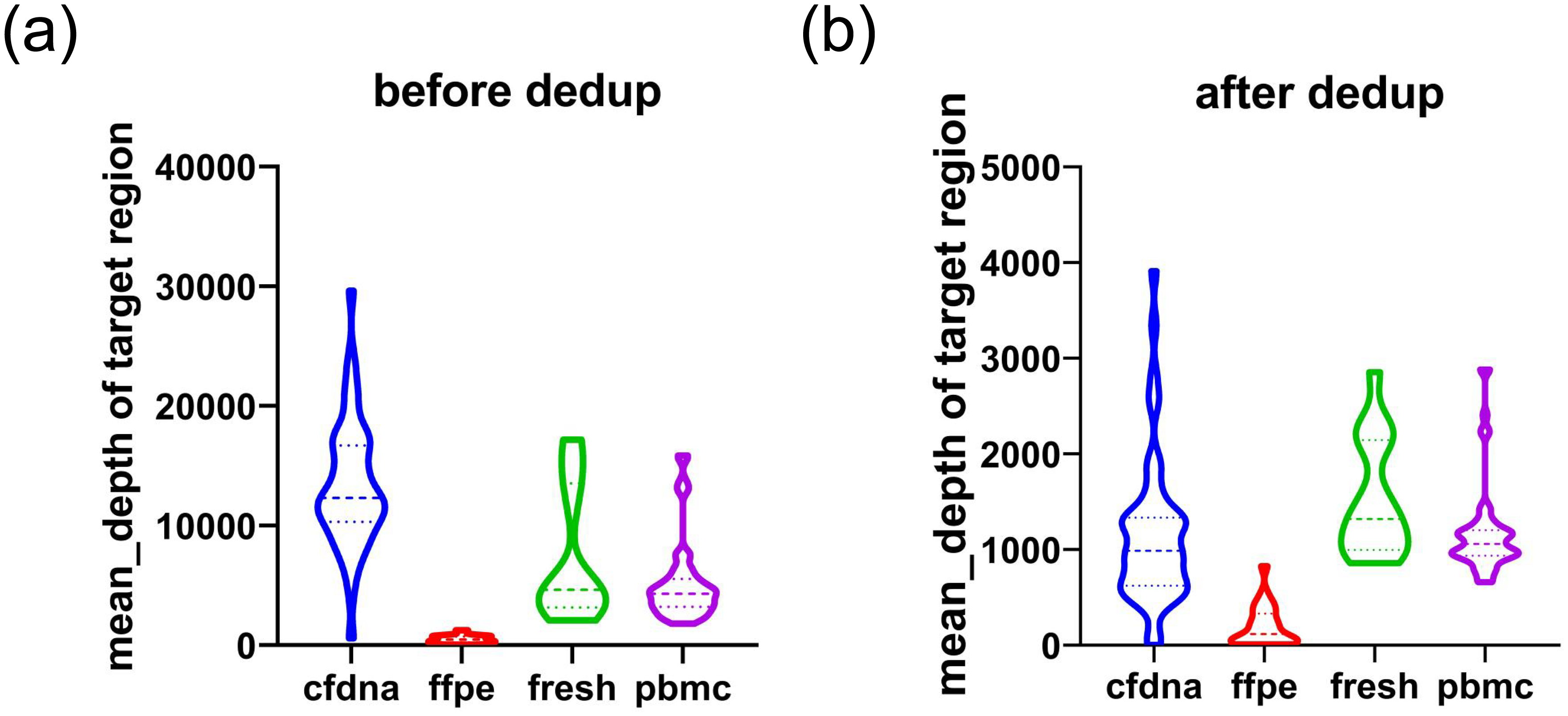

Supplement: S3 Fig — On-target coverage of each sample was plotted before (A) and after (B) deduplication, using the PICARD tool. cfDNA, cell-free DNA; dedup, deduplication; FFPE, formalin-fixed, paraffin-embedded tumor tissue; fresh, fresh-frozen tumor tissue; pbmc, peripheral blood mononuclear cells. (TIFF) [file pone.0232754.s003.tiff]

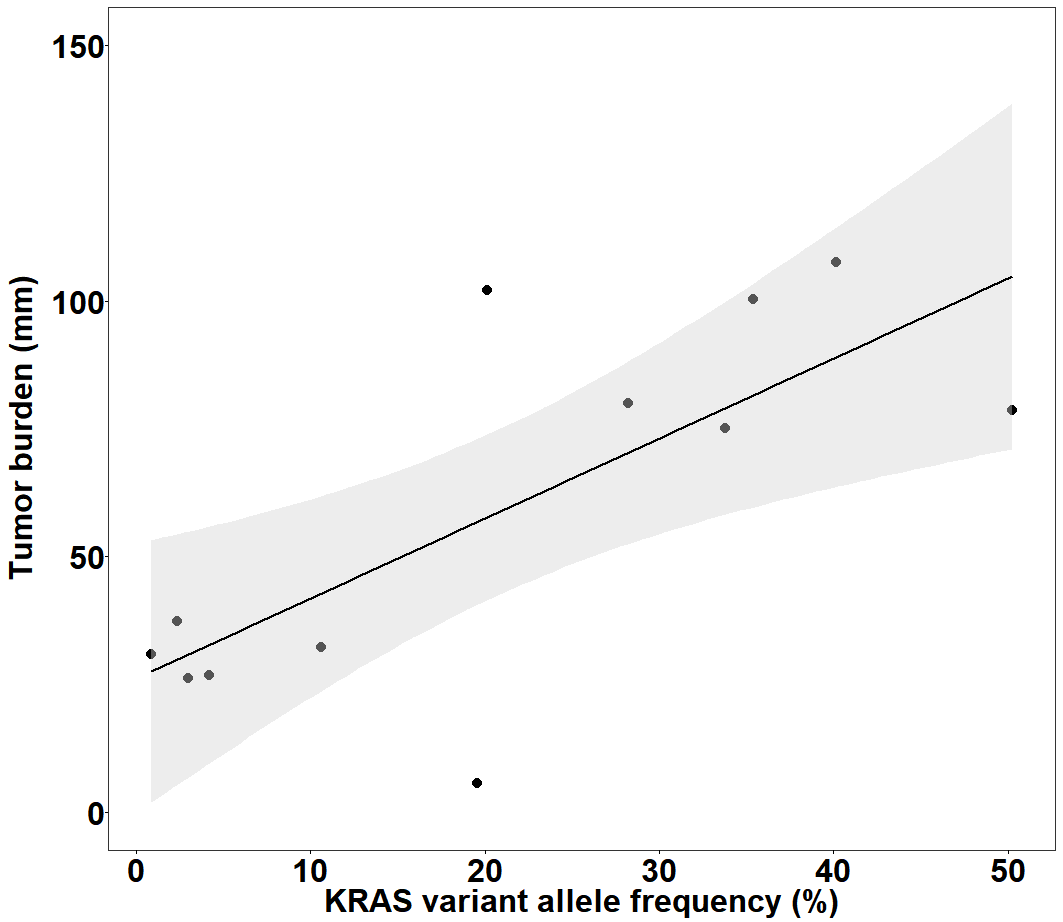

Supplement: S4 Fig — Correlation between KRAS variant allele frequency and total size of liver metastases, excluding patients with disseminated metastatic lesions (n = 12, R2 = 0.55, Spearman’s ρ = 0.69). (TIFF) [file pone.0232754.s004.tiff]

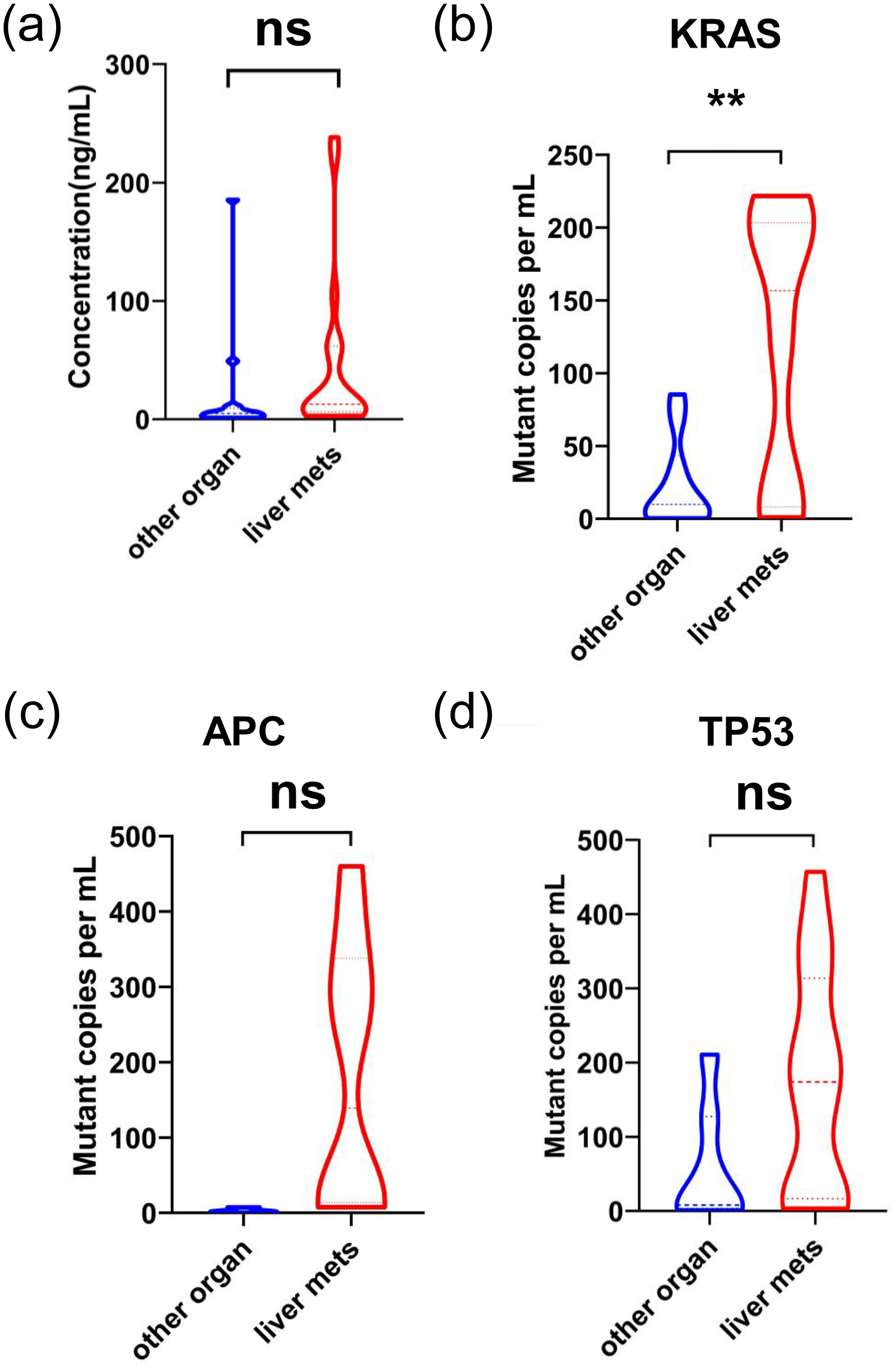

Supplement: S5 Fig — Comparisons between patients with liver metastasis and metastasis in other organs with respect to cfDNA concentration (A) and quantities of specific mutant fragments: KRAS (B), APC (C), and TP53 (D). The quantity of KRAS mutant fragments was significantly higher in patients with liver metastasis (Student’s t-test, p<0.05). ns, not significant. (TIFF) [file pone.0232754.s005.tiff]
